# Lack of Association of P2RX7 Gene rs2230912 Polymorphism with Mood Disorders: A Meta-Analysis

**DOI:** 10.1371/journal.pone.0088575

**Published:** 2014-02-12

**Authors:** Wen-Ping Feng, Bo Zhang, Wen Li, Juan Liu

**Affiliations:** 1 Department of Neurology, the affiliated Hospital of Shangluo Vocational and Technical College, Shangluo, China; 2 Department of Neurology, Central Hospital of Shangluo, Shangluo, China; 3 Department of Neurology, Suzhou BenQ Hospital, the Affiliated Hospital of Nanjing Medical University, Suzhou, China; University of Sao Paulo, Brazil

## Abstract

**Background:**

To assess the association of P2RX7 gene rs2230912 polymorphism with mood disorders using a meta-analysis.

**Methods:**

Data were collected from the following electronic databases: PubMed, Excerpta Medica Database, Elsevier Science Direct, Cochrane Library, and Chinese Biomedical Literature Database, with the last report up to April 1, 2013. Odds ratio (OR) with 95% confidence interval (CI) was used to assess the strength of the association. Dependent on the results of heterogeneity test among individual studies, the fixed effect model (Mantel–Haenszel) or random effect model (DerSimonian–Laird) was selected to summarize the pooled OR.

**Results:**

We identified 13 separate studies using search (6,962 cases and 9,262 controls). We detected significant between-study heterogeneity. No significant association of this polymorphism with mood disorders was found (*P*>0.05). We also performed disease-specific meta-analysis in unipolar depression and bipolar disorder. No significant association of this polymorphism with unipolar depression or bipolar disorder was found (*P*>0.05). Additionally, we performed subgroup analysis by different types of cases. No significant association of this polymorphism with mood disorders in clinical cohorts or population-based cohorts (*P*>0.05). A significant association of this polymorphism with mood disorders was found for the allele contrast in family-based cohorts (OR = 1.26, 95%CI = 1.05–1.50, *P* = 0.01).

**Conclusions:**

Overall, our meta-analysis suggests that P2RX7 gene rs2230912 polymorphism may not contribute to the risk of developing mood disorders using a case-control design. Given the discordance in the subgroup analysis by different types of cases, further studies based on larger sample size are still needed.

## Introduction

Mood disorders define a large group of human psychiatric disorders with a high phenotypic complexity but all characterized by consistent, pervasive alterations in mood, which affect thoughts, emotions and behaviours, and they are among the most prominent causes of disability as well as the second leading source of disease burden [1], [2]. Among them, unipolar disorder and bipolar disorder are two main categories with lifetime prevalence rates of 16% and 1%, respectively [3]. The etiology of mood disorders has not yet been fully described but believed to be multifactorial, and genetic factor plays a role in the pathogenesis of these disorders. Heritability of bipolar disorder is estimated at 90%, whereas for unipolar disorder it is around 40% [4], [5].

The P2X7 receptor is an ATP-gated non-selective cation channel activated by high concentrations of ATP (>100 µM), expressed as homo-oligomeric assemblies of individual subunits, and is widely distributed at immunocompetent cells of the central and peripheral nervous system [6]. Evidence indicates that the P2X 7 receptors may affect neuronal cell death through their ability to regulate the processing and release of interleukin-1β, which is a key mediator in chronic inflammation, neurodegeneration and chronic pain [7]. Moreover, P2X 7 receptor-deficient mice have substantially attenuated inflammatory responses [8]. The excessive secretion of proinflammatory cytokines from activated macrophages has been suggested to play a role in the pathogenesis of mood disorders [9]. Recently, the potential role of P2X 7 receptors in neuronal functions have received considerable attention, and many studies indicate that they are involved in the regulation of diverse neural functions [6]. P2X 7 receptors have been proposed to be potential therapeutic target sites in disorders of the nervous system [6].

P2RX7 (purinergic receptor P2X, ligand-gated ion channel, 7) gene located on chromosome 12q24.31 encodes the P2X7 receptor [10]. The P2RX7 gene was selected as a candidate gene in the first genetic studies of mood disorders based on the results of linkage studies and subsequent detailed studies of the 12q22–24 region [11]–[14]. There are extensive single nucleotide polymorphisms (SNPs) in the P2RX7 gene. Of these SNPs, rs2230912 is located in exon 13 of P2RX7 and results in a change of the amino acid glutamine to arginine at 460 position (Gln460Arg) [15]. In the past six years, a number of studies have investigated the association of this polymorphism with mood disorders, but findings are not always consistent [16]–[25]. There are several possible explanations for this discordance, such as small sample size, ethnic background, different types of mood disorders, and publication bias. Meta-analysis is a statistical procedure for combining the results of several studies to produce a single estimate of the major effect with enhanced precision, and it is considered a powerful tool for summarizing inconsistent results from different studies [26]. The aim of the present study is to perform a comprehensive meta-analysis to evaluate the association between P2RX7 gene rs2230912 polymorphism and mood disorders.

## Methods

### Identification of eligible studies

The present meta-analysis was conducted according to the Preferred Reporting Items for Systematic Reviews and Meta-analysis (PRISMA) guidelines (Checklist S1) [27]. We searched the following electronic databases: PubMed, Excerpta Medica Database (EMBASE), Elsevier Science Direct, Cochrane Library, and Chinese Biomedical Literature Database (CBM). The search stratrgy was based on the key words “P2RX7”, “P2X7”, “affective”, “depression”, “depressive”, “depressed”, “mood” “unipolar”, “bipolar”, “mania”, “manic”, “gene”, “allele”, “polymorphism”, and “variation”. Additional studies were identified by a hand search of references of original studies and review articles on the association between P2RX7 gene polymorphisms and mood disorders. No language restrictions were applied. Abstracts, case reports and editorials were excluded. A study was included in the current meta-analysis if the publications met all of the following criteria: (1) it was published up to April 1, 2013; (2) it was a case-control study of P2RX7 gene rs2230912 polymorphism and mood disorders. When there were multiple publications from the same population, only the largest study was included. When a study reported the results on different subpopulations, we treated them separately. Two reviewers independently searched the electronic databases.

### Data extraction

Two reviewers independently extracted the data with the standard protocol, and the result was reviewed by a third reviewer. Discrepancies were resolved by discussion with our research team. From each study, we extracted the first author, year of publication, journal, ethnicity, types of mood disorders, numbers of cases and controls, and the available genotype and allele frequencies of P2RX7 gene rs2230912 polymorphism. If original data were unavailable in relevant articles, a request for additional data were sent to the corresponding author.

### Statistical analyses

We assessed the relationship between the allele, as well as genotypes and susceptibility to mood disorders. The odds ratio (OR) and its 95% confidence interval (95%CI) were estimated for each study. The heterogeneity between the study results was assessed by the Chi square-test based Q-statistic [28]. A significant Q-statistic (*P*<0.10) indicated heterogeneity across studies. The degree of heterogeneity was further assessed with the *I^2^* statistics (*I^2^* = 100%×(Q-df)/Q) [29]. Meta-regression was performed to detect the source of heterogeneity. Dependent on the results of heterogeneity test among individual studies, the fixed effect model (Mantel–Haenszel) or random effect model (DerSimonian–Laird) was selected to summarize the pooled OR [30], [31]. Publication bias was evaluated by visual inspection of the funnel plot. Funnel plot asymmetry was further evaluated by the method of Egger's linear regression test [32]. Additionally, Chi square-test was used to determine if observed frequencies of genotypes conformed to Hardy-Weinberg equilibrium (HWE) expectations. Analyses were performed using the software Review Manager (v4.2; Oxford, England) and Stata statistical software (v10.0; StataCorp, College Station, TX, USA). *P*<0.05 (two-tailed) was considered statistically significant.

## Results

### Study characteristics

**Figure 1:**
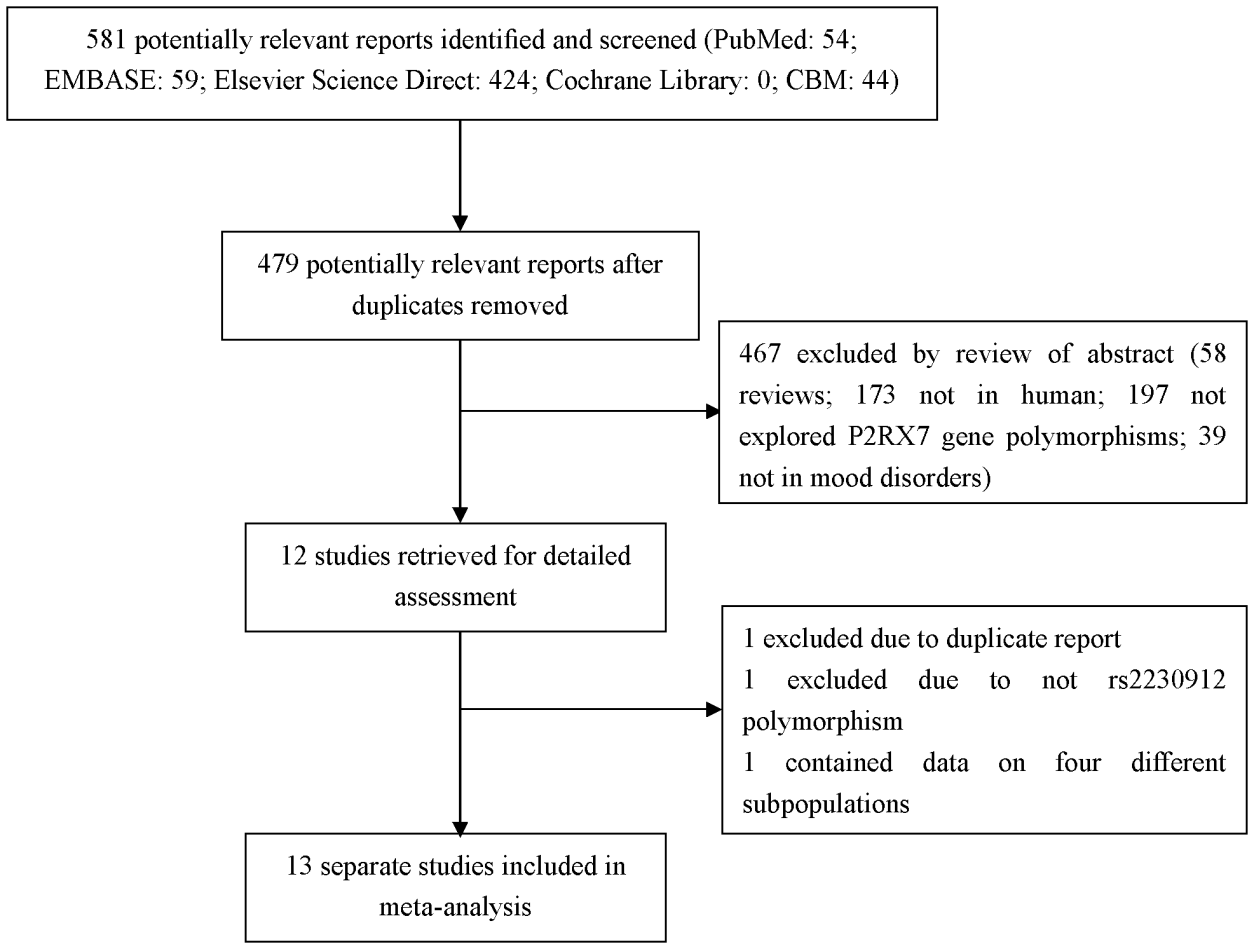
Flow diagram of the study selection process.

The characteristics of studies included in the current meta-analysis are presented in Table 1. The study selection process is shown in Figure 1. There were 581 studies relevant to the searching terms (Pubmed: 54; Embase: 59; Elsevier Science Direct: 424; Cochrane Library: 0; CBM: 44). Totally, 12 studies examined the association between P2RX7 gene polymorphisms and mood disorders [16]–[25], [33], [34]. Of these, 2 studies were excluded (1 was excluded due to duplicate report; 1 was excluded due to not rs2230912 polymorphism) [33], [34]. All of the eligible studies were conducted in Caucasian population. The results of HWE test for the distribution of the genotype in control population are shown in Table 1. The genotype distribution in one study was not in agreement with HWE [24]. It was unavailable for another study to perform HWE test [16]. Of 10 eligible studies, one contained data on four different subpopulations, and we treated them separately [19]. Thus, a total of 13 separate studies were included in the meta-analysis [16]–[25].

**Table 1:** Characteristics of studies investigating the association of P2RX7 gene rs2230912 polymorphism with mood disorders.*

ID	Study (first author, year, reference)	Ethnicity	Types of mood disorders	Types of case	Sample size		Frequencies of genotypes	HWE (*P-value*)	Results
					Case	Control			
1	Soronen 2011 [16]	Caucasian	UD/BD	Family cohorts	450	1322	NA	NA	S
2	Viikki 2011 [17]	Caucasian	UD	Clinical cohorts	218	391	available	>0.05	NS
3	Lavebratt 2010 [18]	Caucasian	UD/dysthymia/mixed anxiety depression	Population cohorts	435	2215	available	>0.05	NS
4	Grigoroiu-Serbanescu 2009 [19]	Caucasian(German/Romanian/Polish/Russian)	UD/BD	Clinical cohorts	2085	2006	available	>0.05	NS
5	Green 2009 [20]	Caucasian	UD/BD	Population cohorts	1710	1179	NA	>0.05	NS
6	Hejjas 2009 [21]	Caucasian	UD/BD	Clinical cohorts	171	178	available	>0.05	NS
7	McQuillin 2009 [22]	Caucasian	BD	Population cohorts	587	546	available	>0.05	S
8	Yosifova 2009 [23]	Caucasian	BD	Clinical cohorts	94	184	NA	>0.05	NS
9	Lucae 2006 [24]	Caucasian	UD	Clinical cohorts	999	1029	available	<0.05	S
10	Barden 2006 [25]	Caucasian	BD	Family cohorts	213	212	available	>0.05	S

*HWE: Hardy–Weinberg equilibrium; UD: unipolar depression; BD: bipolar disorder; NA: not available; S: significant; NS: not significant.

### Meta-analysis results

Table 2 summarizes the main results of this meta-analysis and the heterogeneity test. We detected significant between-study heterogeneity in the contrasts of G versus A, AG+GG versus AA and AG versus AA. No significant association of P2RX7 gene rs2230912 polymorphism with mood disorders was found (*P*>0.05). A sensitivity analysis was performed in those studies fulfilling HWE. The result showed that there was still no significant association of P2RX7 gene rs2230912 polymorphism with mood disorders (data not shown).

**Table 2:** Meta-analysis of P2RX7 gene rs2230912 polymorphism and mood diorders association.*

Comparsions		Sample size		No. of Studies	Test of association				Test of heterogeneity			
		Case	Control		*OR (95%CI)*	*Z*	*P-value*	*Model*	*χ^2^*		*P-value*	*I^2^(%)*
Overall	G vs A	13924	18524	13	1.05(0.96–1.15)	1.04	0.30	R	22.49		0.03	46.6
	AG+GG vs AA	4708	6577	10	1.08(0.94–1.24)	1.09	0.28	R	19.69		0.02	54.3
	GG vs AA+AG	4708	6577	10	0.87(0.68–1.11)	1.12	0.26	F	7.32		0.60	0.0
	GG vs AA	3367	4858	10	0.90(0.70–1.15)	0.85	0.39	F	7.52		0.58	0.0
	AG vs AA	4589	6397	10	1.10(0.95–1.27)	1.27	0.20	R	20.55		0.01	56.2
UD	G vs A	6540	10388	6	1.01(0.87–1.18)	0.17	0.86	R	13.21		0.02	62.2
	AG+GG vs AA	1964	2693	4	0.98(0.76–1.27)	0.14	0.89	R	9.42		0.02	68.1
	GG vs AA+AG	1964	2693	4	0.71(0.49–1.04)	1.77	0.08	F	0.26		0.97	0.0
	GG vs AA	1431	2023	4	0.73(0.50–1.07)	1.62	0.11	F	0.17		0.98	0.0
	AG vs AA	1921	2540	4	0.92(0.63–1.33)	0.44	0.66	R	17.97		0.0004	83.3
BD	G vs A	6514	11254	10	1.08(0.99–1.17)	1.74	0.08	F	13.20		0.15	31.8
	AG+GG vs AA	2309	2942	7	1.09(0.91–1.30)	0.92	0.36	R	11.48		0.07	47.7
	GG vs AA+AG	2309	2942	7	0.99(0.71–1.38)	0.05	0.96	F	5.47		0.48	0.0
	GG vs AA	1629	2149	7	1.02(0.73–1.42)	0.12	0.90	F	5.38		0.44	0.0
	AG vs AA	2242	2857	7	1.10(0.92–1.32)	1.02	0.31	R	11.27		0.08	46.8
Clinical cohorts	G vs A	7134	7576	8	0.96(0.84–1.11)	0.51	0.61	R	14.53		0.04	51.8
	AG+GG vs AA	3473	3604	7	1.00(0.83–1.20)	0.03	0.97	R	16.65		0.01	64.0
	GG vs AA+AG	3473	3604	7	0.78(0.58–1.04)	1.72	0.08	F	2.72		0.84	0.0
	GG vs AA	2510	2674	7	0.79(0.59–1.06)	1.57	0.12	F	2.71		0.84	0.0
	AG vs AA	3389	3492	7	1.02(0.83–1.25)	0.17	0.86	R	18.16		0.006	67.0
Population cohorts	G vs A	5464	7880	3	1.07(0.97–1.18)	1.31	0.19	F	3.24		0.20	38.2
Family cohorts	G vs A	1326	3068	2	1.26(1.05–1.50)	2.55	0.01	F	0.01		0.99	0.0

*UD: unipolar depression; BD: bipolar disorder; vs: versus; R: random effect model; F: fixed effect model.

We also performed disease-specific meta-analysis in unipolar depression (six studies, 3,270 cases and 5,194 controls) and bipolar disorder (ten studies, 3,257 cases and 5,627 controls). In the analysis of unipolar depression, significant between-study heterogeneity was found in the contrasts of G versus A, AG+GG versus AA and AG versus AA. We did not detect significant association of P2RX7 gene rs2230912 polymorphism with unipolar depression (*P*>0.05). In the analysis of bipolar disorder, significant between-study heterogeneity was found in the contrasts of AG+GG versus AA and AG versus AA. Similarly, no significant association of P2RX7 gene rs2230912 polymorphism with bipolar disorder was found (*P*>0.05).

Additionally, we performed subgroup analysis by different types of cases (clinical cohorts: eight studies including 3,567 cases and 3,788 controls; population-based cohorts: three studies including 2,732 cases and 3,940 controls; family-based cohorts: two studies including 663 cases and 1,534 controls). Significant between-study heterogeneity was found for the contrasts of G versus A, AG+GG versus AA and AG versus AA in clinical cohorts, but not for other contrasts. No significant association of this polymorphism with mood disorders in clinical cohorts or population-based cohorts (*P*>0.05). A significant association of this polymorphism with mood disorders was found for the allele contrast in family-based cohorts (OR = 1.26, 95%CI = 1.05–1.50, *P* = 0.01).

### Evaluation of publication bias and heterogeneity

Funnel plot and Egger's linear regression test were performed to estimate the publication bias of literatures. The shapes of the funnel plots did not reveal any evidence of obvious asymmetry (funnel plots not shown). The results of Egger's linear regression test are shown in Table 3. The intercept *a* provides a measure of asymmetry, and the larger its deviation from zero the more pronounced the asymmetry. It was shown that there was no publication bias for most of comparisons. However, Egger's linear regression test was not applied for some comparisons due to the small number of studies. As shown in Table 2, significant heterogeneity was found, thus we performed meta-regression analysis for predefined underlying sources of heterogeneity, including year of publication, region, types of mood disorders, types of cases and sample size. Meta-regression indicated that types of cases (*P* = 0.051) may contribute to heterogeneity.

**Table 3:** Egger's linear regression test to measure the funnel plot asymmetric.*

Comparsions	Y axis intercept: *a (95%CI)*				
	G vs A	AG+GG vs AA	GG vs AA+AG	GG vs AA	AG vs AA
Overall	−1.33(−3.63–0.96)	−1.91(−5.08–1.25)	0.02(−2.48–2.54)	−0.20(−2.74–2.32)	−1.89(−5.12–1.32)
UD	−1.37(−7.46–4.72)	−3.58(−10.99–3.83)	0.23(−2.01–2.48)	−0.21(−2.04–1.61)	−3.67(−11.91–4.56)
BD	−1.79(−4.16–0.56)	−1.74(−5.98–2.50)	−1.46(−5.22–2.29)	−1.66(−5.44–2.11)	−1.45(−5.71–2.79)
Clinical cohorts	−2.48(−5.06–0.08)	−2.72(−6.58–1.14)	0.26(−2.81–3.35)	−0.33(−2.61–1.93)	−2.77(−6.82–1.28)
Population cohorts	4.92(−12.73–22.58)	–	–	–	–

*All *P*>0.05; UD: unipolar depression; BD: bipolar disorder; vs:versus.

## Discussion

There is compelling evidence from family, twin and adoption studies for the existence of genes influencing susceptibility to mood disorders [4], [5]. The enormous public health importance of mood disorders has stimulated much work aimed at identifying susceptibility genes. Association studies in mood disorders over recent years have focussed attention on several candidate genes [35]. However, findings are not always consistent. Large samples of subjects are necessary to obtain suffcient power of detection [36]. Small sample sized association studies lack statistical power and may result in contradicting findings [37]. Meta-analysis has become important in psychiatric disorders because of rapid increases in the number and size of datasets. In the meta-analysis, we retrieved 10 studies that included data from 6,962 cases and 9,262 controls to evaluate the association between P2RX7 gene rs2230912 polymorphism and mood disorders. Overall, we did not find significant association of this polymorphism with mood disorders. To our knowledge, the present meta-analysis is the first to assess the association between P2RX7 gene rs2230912 polymorphism and mood disorders.

In the central and peripheral nervous system, the P2X7 receptors are expressed on microglial cells, neurons and astrocytes. They take part in inflammatory responses and the cross-talk between glia and neurons, and are proposed to promote neurotransmitter release at presynaptic sites [7]. The P2X7 receptors may have a role to play in susceptibility to mood disorders. Genetic linkage studies have previously identified many single non-synonymous nucleotide polymorphisms in the human P2RX7 gene in individuals with mood disorders. The non-synonymous SNP rs2230912 is located in the C-terminal domain of the P2X7 ion channel, suggesting a role in receptor function, and protein-protein or protein-lipid interaction [38]. However, there is some controversy concerning the functionality of rs2230912, and, recently, a well-conducted study showed that this polymorphism did not give rise to profound effects on the P2X7 receptors function [39]. In the present study, we found no support for the specific hypothesis that P2RX7 gene rs2230912 polymorphism influences susceptibility to mood disorders in overall analysis and disease-specific analysis. Cases from clinical cohorts, population-based cohorts and family-based cohorts may have different clinical pictures of mood disorders. In the subgroup analysis by different types of cases, no significant association of this polymorphism with mood disorders was found in clinical cohorts or population-based cohorts. A significant association of this polymorphism with mood disorders was found for the allele contrast in family-based cohorts. However, in family-based cohorts, only two studies were included the meta-analysis, and further studies based on larger sample size are still needed to verify the finding. Additionally, molecular biological studies are required to identify the functional importance of this polymorphism on receptor function, and neurobiological investigations are needed to shed light on the connection between P2X7 receptor functioning and mood disorders.

Several specific details merit consideration in the current meta-analysis. A first consideration is that only published studies were included in this meta-analysis, and Egger's linear regression test was not applied for some comparisons due to the small number of studies. Thus, publication bias may occur. A second consideration is that significant between-study heterogeneity was detected in some comparisons, and may be distorting the results of the meta-analysis. Further studies based on refined phenotypes may help to elucidate the association of P2RX7 gene rs2230912 polymorphism with mood disorders. A third consideration is that our reulsts are based on unadjusted estimates, and a more precise analysis stratified by age and sex could be performed if individual data were available. Finally, all of the eligible studies were conducted in Cauasian population. Further studies are still needed in non-Cauasian population.

Overall, our meta-analysis suggests that P2RX7 gene rs2230912 polymorphism may not contribute to the risk of developing mood disorders using a case-control design. Given the discordance in the subgroup analysis by different types of cases, further studies based on larger sample size are still needed.

## Supporting Information

Checklist S1 **PRISMA checklist.**
(DOC)
